# THz Electric Field-Induced Second Harmonic Generation in Inorganic Ferroelectric

**DOI:** 10.1038/s41598-017-00704-9

**Published:** 2017-04-06

**Authors:** Kirill A. Grishunin, Nikita A. Ilyin, Natalia E. Sherstyuk, Elena D. Mishina, Alexey Kimel, Vladimir M. Mukhortov, Andrey V. Ovchinnikov, Oleg V. Chefonov, Mikhail B. Agranat

**Affiliations:** 1 Moscow Technological University, MIREA, Vernadsky Ave. 78, 119454 Moscow, Russia; 2 Institute for Molecules and Materials, Radboud University Nijmegen, 6525 AJ Nijmegen, The Netherlands; 3 Southern Scientific Center of Russian Academy of Sciences, Chehova 41, Rostov-on-Don, 344006 Russia; 4 Joint Institute for High Temperatures of Russian Academy of Sciences (JIHT), Izhorskaya st. 13 Bd. 2, 125412 Moscow, Russia

## Abstract

Second Harmonic Generation induced by the electric field of a strong nearly single-cycle terahertz pulse with the peak amplitude of 300 kV/cm is studied in a classical inorganic ferroelectric thin film of (Ba_0.8_Sr_0.2_)TiO_3_. The dependences of the SHG intensity on the polarization of the incoming light is revealed and interpreted in terms of electric polarization induced in the plane of the film. As the THz pulse pumps the medium in the range of phononic excitations, the induced polarization is explained as a dynamical change of the ferrolectric order parameter. It is estimated that under action of the THz pulse the ferroelectric order parameter acquires an in-plane component up to 6% of the net polarization.

## Introduction

Photoinduced nonequilibrium phase transitions triggered by femtosecond or picosecond laser pulses is a subject of intense and multidisciplinary research^1, 2^. Fundamentally, these are counterintuitive phenomena in which subtle excitations of atoms or spins are able to lead to dramatic changes in crystallographic, electric, or magnetic properties of media. In ferroics, ultrafast control of the order parameter (electric polarization or magnetization) is a particularly important problem, understanding of which might be essential for future progress in information processing technology. In the last decades a substantial progress has been achieved in ultrafast optical control of spins in magnetically ordered materials and all-optical magnetic recording. It has been shown that exciting magnets on a timescale much faster than characteristic times of atomic, orbital and spin motion can steer magnetization dynamics along yet unexplored non-thermodynamic routes^3^. Ultrafast coherent control of the magnetic phase transitions via active optical pumping of the soft mode^4, 5^ and magnetization reversal via a strongly non-equilibrium state^6^ have been demonstrated. Thermodynamically, critical behaviors of the electric polarization and the magnetization are very similar^7^. Nevertheless, the possibility of switching of the electric polarization by ultrashort laser pulses has not been reported until now. Naturally it raises interests to the problem of ultrafast optical control of electric polarization in ferroelectric materials.

Despite the enormous amount of experiments reporting on optical control of spins, there are very few studies of ultrafast dynamics of the electric polarization^8–16^. Obviously, measurement techniques based on electric contacts are not able to provide the required temporal resolution. An elegant solution for a detection of the order parameter in ferroelectrics can be based on time-resolved X-ray diffraction. However, this technique is very challenging and, as a matter of fact, it is available in very few places in the world^17–19^. Nonlinear optical technique of the Second Harmonic Generation (SHG) is known to be very sensitive to the order parameter in ferroelectrics^16, 20–22^ and it is by far less challenging than the studies in the X-ray spectral range^23–25^. However, despite this fact practically nothing is known about ultrafast nonlinear optical response of ferroelectrics^26^. Consequently, this lack of knowledge considerably hampers optimization and interpretation of experiments in which ultrafast dynamics in ferroics is probed with the help of second harmonic generation and visible light.

Here we report about experimental study of ultrafast nonlinear optical response of ferroelectric (Ba_0.8_Sr_0.2_)TiO_3_ (BST) to the electric field of nearly single-cycle THz pulses picosecond pulses. For this we employ a pump-probe method. Freely propagating nearly single-cycle THz pulses with the amplitude up to 300 kV/cm excites ferroelectric ((Ba_0.8_Sr_0.2_)TiO_3_) film. Femtosecond pulses in the near-infrared spectral range probe the response of the system by SHG. Analysis of the time-resolved non-linear optical response reveals that the experimental results can be explained assuming that under action of the THz pulse the ferroelectric order parameter acquires an in-plane component up to 6% of the net polarization.

## Results

**Figure 1:**
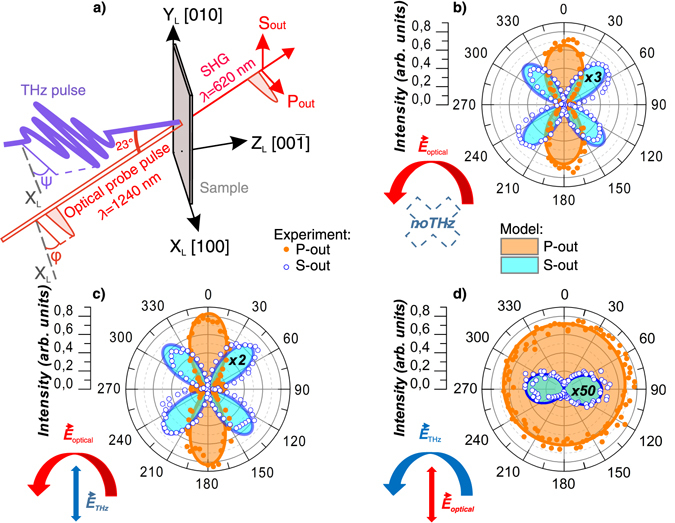
Experimental geometry and polarization diagrams of the SHG intensity for various experimental geometries. (**a**) Experimental geometry. The axes of the chosen laboratory frame X_L_, Y_L_, Z_L_ correspond to [100], [010] and [${\rm{00}}\overline{{\rm{1}}}$] crystallographic directions, respectively. *φ* - the angle between the electric field of near-infrared probe and the X_L_-axis. *ψ* - the angle between the electric field of the THz pump pulse and the X_L_-axis; (**b**) dependence of the SHG signal on *φ* without any THz pump; (**c**) dependence of the SHG signal on *φ* when the THz field is applied parallel with respect to the X_L_-axis. The polarization of the SHG signal was set either in the P_out_ or S_out_-state; (**d**) dependence of the SHG signal on *ψ* when the probe polarization was set to the P-state (*φ* = 0). The polarization of the SHG signal was set either in the P_out_ or S_out_-state. Dots correspond to experimental data and lines are fits (calculations see in Supplementary Information). Values for S-state multiplied by 3, 2 and 50 for (**b**–**d**) respectively.

We have used single-cycle THz pulses to excite the ferroelectric. The result of the excitation was probed by a femtosecond laser with the central wavelength of 1240 nm. Upon non-linear interaction of this pulse with the ferroelectric medium, second harmonic light was generated with the central wavelength of 620 nm. The time delay *τ*_d_ between the THz-pump and optical-probe pulses was changed with the help of a delay line. The pump beam was at normal incidence. The angle of incidence of the probe beam was 23 degrees. Schematics of the experimental setup is shown in Fig. 1a. In the following orientation of the electric fields of the pump and probe pulses will be described in laboratory frame with X_L_,Y_L_ and Z_L_ axes, as shown in the figure. The sample was oriented with its [100] axis parallel to the X_L_ axis. The electric field vector of the THz pump pulse was oriented in the X_L_Y_L_ plane at angle *ψ* with respect to the X_L_ axis. The electric field (i.e. polarization) of the near-infrared probe pulse could be rotated with respect to the X_L_ axis by angle *φ* or set in the P-state (*φ* = 0). The polarization of the second harmonic light was chosen either in the P- or S-states of incidence (P_out_ or S_out_ in Fig. 1a, respectively).

In the first experiment, time-delay between pump and probe pulses *τ*_d_ was set to zero. The detected SHG signal of the P_out_ and S_out_ polarizations was studied as a function of the probe polarization angle *φ*. With no THz electric field, the SHG intensities reveal two-fold and four-fold polarization dependencies (see Fig. 1b). If the strong THz field is applied to the ferroelectric material and *ψ* = 0, the intensity of the SHG signal slightly increases, but the polarization dependences remain qualitatively the same (Fig. 1c).

In the second experiment, we fixed the polarization of the near-infrared probe pulse in the P-state and rotated the electric field of the THz pulse changing *ψ*. If the polarization of the outcoming second harmonic light is in the P_out_-state, the SHG intensity only slightly depends on *ψ*. For the S_out_-state of the SHG signal, the dependence is twofold with the maxima when the THz field is along the X_L_ axis (*ψ* = 0).

**Figure 2:**
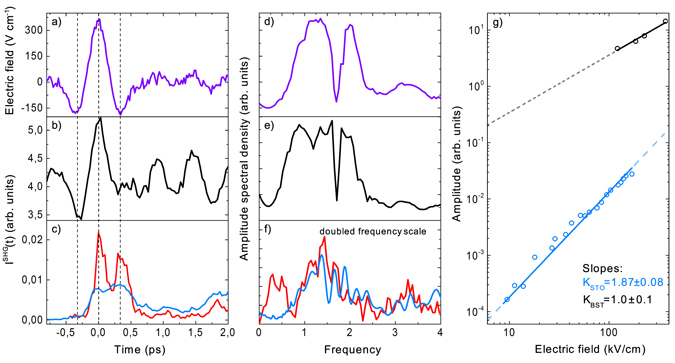
THz-induced dynamics of nonlinear-optical response of the crystals. (**a**) Time trace of the electric field of the THz pump pulse; intensities of the SHG-signal: (**b**) for BST, (**c**) for STO (red line) and Si (black line); (**d**–**f**) Fourier transform spectra of the time traces shown in panels (**a**–**c**), respectively ((**f**) plotted in doubled frequency scale); (**g**) dependence of the SHG intensity on the THz field (logarithmic scale) for BST (top line) and STO (bottom line).

Hence the data show that the THz electric field clearly affects the process of the second harmonic generation. To reveal ultrafast dynamics of these electric field induced changes, we performed pump-probe measurements of the SHG signal. In particular, the signal was measured as a function of the delay *τ*_d_ between the THz-pump and near-infrared probe pulses. Figure 2a shows time-domain trace of the electric field of the THz pulse obtained with the help of electro-optical sampling. The measurements of the SHG signal from the BST film (Fig. 2b) reveal a similar dynamics during the overlap of the probe and THz-pump pulses. It points out that the non-linear response is proportional to the THz electric field.

For comparison, we also measured temporal evolution of the SHG signals from centrosymmetric crystals excited by the intense THz pulse. The SHG transients for SrTiO_3_ (STO) and Si are shown in Fig. 2c (red and blue line, respectively). First of all, for the both cases of the centrosymmetric crystals the SHG signal of the unperturbed media are zero. Secondly, in the maximum of THz modulated signal the absolute values of the intensity of the SHG from BST is two orders of magnitude higher than those for the centrosymmetric crystals. Thirdly, the shape of the SHG response differs from the shape of the THz pulse. Qualitatively, the frequency of the time transients of the SHG response for centrosymmetric STO and Si is doubled with respect to the frequency of the temporal variations of the electric field of the THz pulse.

The spectra obtained as Fourier transforms of the time-traces for BST and STO as well as for THz pulse are plotted in Fig. 2d and e. For BST, the frequency-domain signal follows the input pulse, while for STO the frequency is doubled. Power dependencies of the SHG intensity on the THz electric field are plotted in Fig. 2f in logarithmic scale and reveal linear and quadratic for BST and STO, respectively.

**Figure 3:**
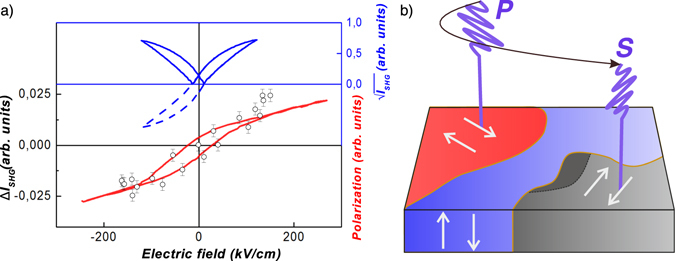
Effect of the electric field on electric polarization and SHG. (**a**) Hysteresis loops of the electric polarization obtained by the Soyer-Tower technique (bottom solid line); SHG hysteresis loop reconstructed from low-frequency measurements (top line). To compare with the hysteresis loop for the electric polarization, left side of the graph is plotted with an inversed sign (dashed line); SHG loops reconstructed from the experiment with THz pump pulses (points). Error bars on ΔI_SHG_ show s.d. from the 15 measured samples; (**b**) diagram showing the domain structure and its sensitivity to the external THz pulse.

Finally, from the measured SHG traces we deduced how the induced electric polarization changes upon a change of the electric field of the THz pulse. These dependencies were compared with the hysteresis loops measured with the help of the Soyer-Tower technique (Fig. 3a, bottom solid line). The first, i.e. static hysteresis loop, is quite typical for BST films with 20% of Strontium^27^. We also compared the data with loops obtained by measuring the SHG signal in an electric field which varies with the frequency of 100 Hz. To deduce the loops we employed the procedure of reconstruction of the electric polarization as described previously^28^. It is based on the dependence of SHG intensity on ferroelectric polarization:

1 $${{\rm{I}}}^{2\omega }={{\rm{I}}}_{{\rm{b}}{\rm{g}}}^{2\omega }+\alpha {({{\rm{P}}}_{0}+{\rm{P}}({{\rm{E}}}_{{\rm{\Omega }}}))}^{2},$$

where ${{\rm{I}}}_{{\rm{bg}}}^{{\rm{2}}\omega }$ is the incoherent component of the unswitchable part of the second harmonic signal; P(E_Ω_) is the ferroelectric (switchable) polarization, which depends on the electric field of the THz pulse; P_0_ is remanent polarization i.e. unswitchable polarization; *α* is the proportionality coefficient, which is determined by the Fresnel factors and the nonlinear optical susceptibility.

In order to deduce the field dependence of the electric polarization P(E_Ω_), one has to subtract from the SHG intensity the background signal ${{\rm{I}}}_{{\rm{bg}}}^{{\rm{2}}\omega }$ which is independent on the electric field. Then one has to take the square root from the residual signal. It gives the value, which is proportional to the electric polarization P_0_ + P(E_Ω_). The result of such a data processing is shown in Fig. 3a (top solid line). Additionally, we can mirror the loop with respect to the x-axis at negative electric fields and thus obtain a loop that resembles largely the dielectric hysteresis loop obtained in statics (Fig. 3a, dashed line). Since in the THz measurements both electric field and SHG intensity are measured as a function of time, hysteresis loop is set parametrically. After excluding time from these dependences and performing the same procedure for the SHG intensity (except taking the square root because of linear dependence of the SHG intensity), the THz SHG loop is obtained. In Fig. 3a points show hysteresis loops for two values of the THz electric field (E-field). Although these loops are quite noisy, a similarity with the low-frequency loop (with hysteresis and coercively) is obvious.

## Discussion

In centrosymmetric crystals (SrTiO_3_, Si), in the electric-dipole approximation, it is possible to generate second harmonic signal by applying an external electric field, which breaks the inversion symmetry. This is so-called electric field induced second harmonic (EFISH). For electric field oscillating at THz frequency we will call it TEFISH. Taking into account that Ω ≪ *ω* and Ω + *ω* + *ω* ≅ 2*ω*, TEFISH polarization can be described as ${\displaystyle \overrightarrow{{\rm{P}}}}^{{\rm{T}}{\rm{E}}{\rm{F}}{\rm{I}}{\rm{S}}{\rm{H}}}(2\omega )={\hat{\chi }}^{(3)}{\displaystyle \overrightarrow{{\rm{E}}}}_{{\rm{\Omega }}}{\displaystyle \overrightarrow{{\rm{E}}}}_{\omega }{\displaystyle \overrightarrow{{\rm{E}}}}_{\omega }$. Analogously to the linear optical Kerr effect, which is described by a tensor of the same rank, it has electronic and ionic contributions^29^.

In noncentrosymmetric crystals with nonzero electric dipole contribution ${\displaystyle \overrightarrow{{\rm{P}}}}^{{\rm{c}}{\rm{r}}{\rm{y}}{\rm{s}}{\rm{t}}}(2\omega )$, formally the same electric field induced mechanism is also valid:

2 $$\overrightarrow{{\rm{P}}}(2\omega )={\overrightarrow{{\rm{P}}}}^{{\rm{c}}{\rm{r}}{\rm{y}}{\rm{s}}{\rm{t}}}(2\omega )+{\overrightarrow{{\rm{P}}}}^{{\rm{T}}{\rm{E}}{\rm{F}}{\rm{I}}{\rm{S}}{\rm{H}}}(2\omega )={\hat{\chi }}^{(2)}{\overrightarrow{{\rm{E}}}}_{\omega }{\overrightarrow{{\rm{E}}}}_{\omega }+{\hat{\chi }}^{(3)}{\overrightarrow{{\rm{E}}}}_{{\rm{\Omega }}}{\overrightarrow{{\rm{E}}}}_{\omega }{\overrightarrow{{\rm{E}}}}_{\omega }.$$

For ferroelectric material electric field dependent nonlinear optical polarization has several contributions (analogously to linear optics^29–31^): electronic, ionic and piezoelectric. As the employed THz pulses pump the medium in the range of phononic excitations, it is natural to assume that the THz electric field induces ferroelectric polarization due to the ionic contribution.

Without any electric field, the sample is split into two types of domains with the polarization aligned along the [001] axis. For nonzero angle of incidence, for the SHG intensity one finds ${{\rm{I}}}_{2\omega }\sim {({{\rm{P}}}_{2\omega }^{001})}^{2}$.

When the in-plane electric field is applied at angle *ψ* respect to X_L_ axis, a part of the domains line up along the field. In analogy with ref. 32, the net response will be defined by volume fractions of domains ${{\rm{V}}}_{{\rm{i}}}^{+}$ and ${{\rm{V}}}_{{\rm{i}}}^{-}$(i = x, y) (i = x, y), where x, y denote the crystallographic axis along which the polarization is aligned, but “+” and “−” show the direction of the alignment. The differences of the fractions of the positively and the negatively oriented domains determines the electric field dependent contribution to the nonlinear optical polarization as ${{\rm{P}}}_{{\rm{i}}}={{\rm{P}}}_{{\rm{i}}}^{001}+{{\rm{\Delta }}{\rm{V}}}_{{\rm{i}}}{{\rm{P}}}_{{\rm{i}}}$, where ${{\rm{\Delta }}{\rm{V}}}_{{\rm{i}}}={{\rm{V}}}_{{\rm{i}}}^{+}-{{\rm{V}}}_{{\rm{i}}}^{-}$.

Thus, the volume contributions to the corresponding domain directions for any angle *ψ* of the applied THz E-field results in the following dependences:

3 $${\rm{\Delta }}{{\rm{V}}}_{{\rm{x}}}=\gamma \cos \psi ,$$

4 $${\rm{\Delta }}{{\rm{V}}}_{{\rm{y}}}=\gamma \sin\psi ,$$

5 $${\rm{\Delta }}{{\rm{V}}}_{{\rm{z}}}=\sqrt{1-{\rm{\Delta }}{{{\rm{V}}}_{{\rm{x}}}}^{2}-{\rm{\Delta }}{{{\rm{V}}}_{{\rm{y}}}}^{2}},$$

where *γ* is the ratio of the fraction of in-plane switched domains to the fraction of [001]-oriented unswitched domains.

Generally, SHG intensity for the THz E-field oriented along arbitrary direction in the plane of the sample can be written as

6 $${{\rm{I}}}_{2\omega }\propto {({{\rm{P}}}_{2\omega }^{001}{\rm{\Delta }}{{\rm{V}}}_{{\rm{z}}}+{{\rm{P}}}_{2\omega }^{100}({{\rm{E}}}_{{\rm{\Omega }}}){\rm{\Delta }}{{\rm{V}}}_{{\rm{x}}}+{{\rm{P}}}_{2\omega }^{010}({{\rm{E}}}_{{\rm{\Omega }}}){\rm{\Delta }}{{\rm{V}}}_{{\rm{y}}})}^{2}.$$

When the in-plane electric field is applied along the [100] axis (V_x_ ≠ 0, V_y_ = 0), a polarization is induced along the same axis and the intensity acquires an additional contribution:

7 $${{\rm{I}}}_{2\omega }\propto {({{\rm{P}}}_{2\omega }^{001}{\rm{\Delta }}{{\rm{V}}}_{{\rm{z}}})}^{2}+2{{\rm{P}}}_{2\omega }^{001}{{\rm{P}}}_{2\omega }^{100}({{\rm{E}}}_{{\rm{\Omega }}}){\rm{\Delta }}{{\rm{V}}}_{{\rm{z}}}{\rm{\Delta }}{{\rm{V}}}_{{\rm{x}}}+{({{\rm{P}}}_{2\omega }^{100}({{\rm{E}}}_{{\rm{\Omega }}}){\rm{\Delta }}{{\rm{V}}}_{{\rm{z}}})}^{2}.$$

Analogously, similar dependeces of the SHG intensity can be written for the case of THz field, oriented along the y axis. Such a field promotes formation of ΔV_y_ domains.

The results based on the suggested model show a good agreement with the experimental data (Fig. 1). In the simultaneous fit only 2 meaningful and 1 calibration fitting parameters were used giving *χ*_1_/*χ*_2_ = 3.4 ± 0.3 for the wavelength of the probing light of 1240 nm (compare *χ*_1_/*χ*_2_ = 2.6 ± 0.4 for the wavelength of 1058 nm^33^ for BaTiO_3_) and *γ* = 0.06 (calculations for the cases represented on Fig. 1 see in Supplementary Information).

**Figure 4:**
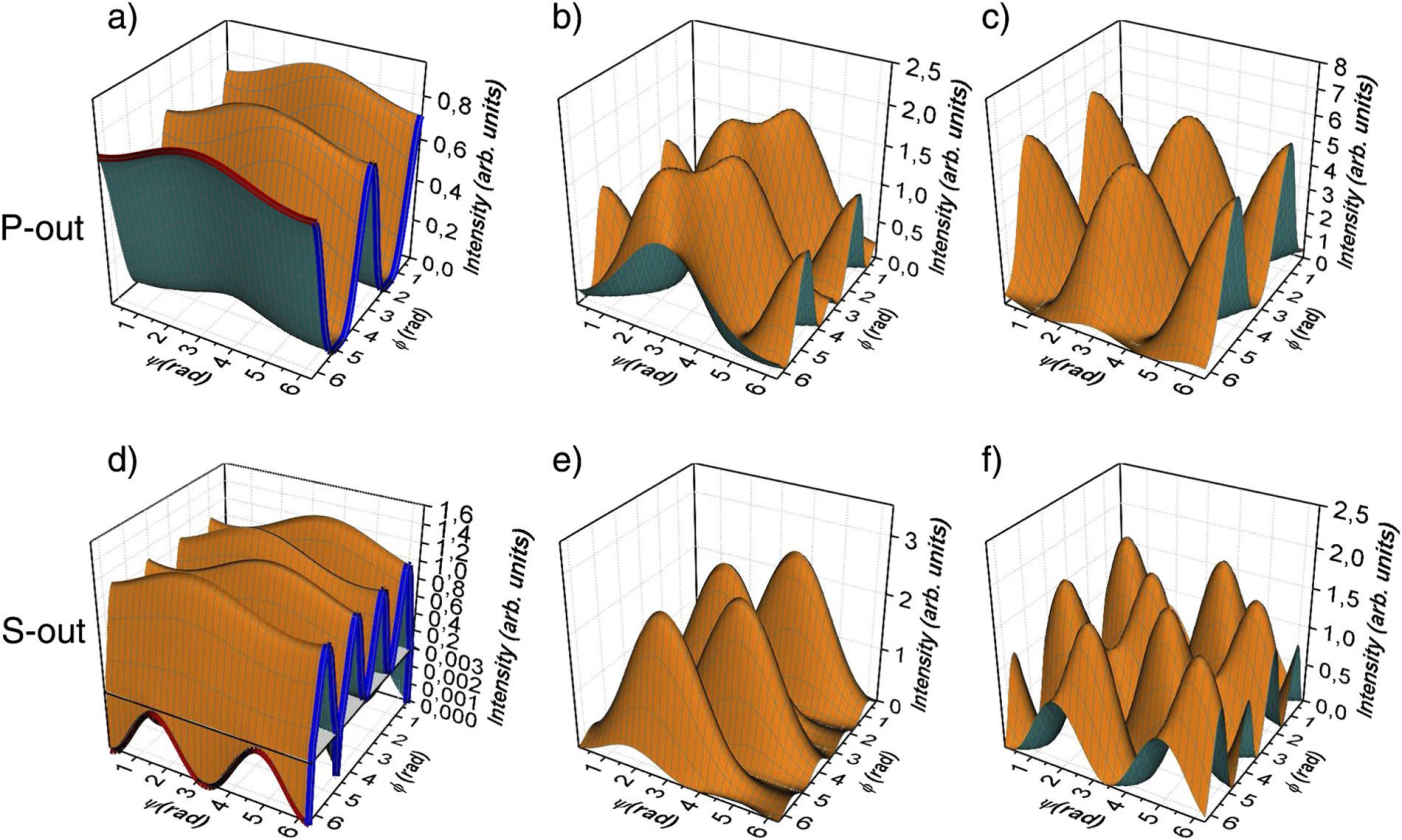
Polarization dependences obtained within the suggested model for different *γ*: (**a**,**d**) *γ* = 0.05; (**b**,**e**) *γ* = 0.5; (**c**,**f**) *γ* = 1. Blue and red lines on (**a** and **d**) represent the linear plots of fitting curve from Fig. 1c and d.

Figure 4 shows examples of polarization dependences obtained for different *γ*. An increase of *γ* results in an increase of the SHG intensity and changes in polarization dependences. When the THz electric field is rotated, asymmetry of one-fold I_2*ω*_(*ψ*) first increases (Fig. 4b and e), but then for *γ* → 1 the dependences acquire a two-fold symmetry. When optical polarization is rotated, four-fold I_2*ω*_(*φ*) dependence for the S_out_ state of the SHG electric field changes only in intensity. For the P_out_ state, the two-fold I_2*ω*_(*φ*) dependence changes passing through four-fold dependence. Cross-sections of the 3D plots along the planes (blue and red lines on Fig. 4a and d) represent the linear plots of the fitting curve from Fig. 1c and d, respectively.

The question arises: can we really switch out-of-plane domains by an in-plane electric field? Very recently it was shown that in classical ferroelectric Pb(Zr_0.4_Ti_0.6_)O_3_ domain nucleation time under electric field of 200 kV/cm was about 0.47 ps^34^. The strength of the electric field required for the switching and nucleation time are very close to the amplitude and the period of the THz electric field used in our experiments. Thus, it seems that in our experiment a switching of the 90-degrees ferroelectric domains, as schematically shown in Fig. 3b, may indeed take place.

## Conclusions

In our experiments with ferroelectric thin film, we observed a very strong modulation of the SHG signal by the electric field of a THz nearly single cycle pulse. Polarization dependences of the SHG intensity were explained in terms of THz electric field-induced second harmonics generation THz EFISH. As the THz pulse pumps the medium in the range of phononic excitations, the modulation of the nonlinear signal can be explained in terms of a change of the ferrolectric order parameter. In particular, under action of the THz pulse the latter acquires an in-plane component up to 6% of the net polarization.

The net SHG signal responds to the THz excitation as if the THz pulse induces a 90-degrees switching of the polarization in parts of the sample. The possibility of such a switching on the time scale of the period of the soft mode was recently suggested by ref. 34.

## Methods

### Samples

Heteroepitaxial (Ba_0.8_Sr_0.2_)TiO_3_ (BST) thin film (thickness 500 nm) was deposited on MgO (001) substrate by RF-sputtering of stoichiometric polycrystalline target. Transparent and mirror-smooth film was realized by means of layer-by-layer growth (Frank-van der Merwe mechanism). Details of the growth conditions have been previously reported in ref. 35. The vertical and azimuthal film misorientations were found to be less than 0.4° by high resolution XRD analysis. (Ba_0.8_Sr_0.2_)TiO_3_ solid solutions belong to ferroelectric perovskites. In the paraelectric phase, they have a cubic unit cell (space group Pm3m). In the ferroelectric phase below the Curie temperature (Tc = 353 K they have a tetragonal unit cell (space group P4mm, point group 4mm). At room temperature, the soft mode is overdamped with the following parameters: Ω = 46 cm^−1^ (2.40 THz), ΔΩ = 50 cm^−1^ (1.5 THz)^36, 37^. The as-grown film consists of 180-degrees domains which are not compensated due to an interfacial strain and show built-in polarization in the [001] crystallographic direction^37, 38^. The size of domains is about 500 nm.

### Experimental setup

The THz pulses were generated by optical rectification of the femtosecond laser pulses, generated by high-energy 10 Hz Cr:Forsterite chirped-pulse amplification laser at the central wavelength of 1240 nm with 100 fs pulse duration and 20 mJ output energy, in the nonlinear organic crystal OH1^39^. The polarization of the terahertz pulse coincided with the polarization of the femtosecond pump laser pulse. The rotation of the terahertz electric field was carried out by simultaneous rotation of polarization of incident beam and nonlinear organic crystal OH1 on the same angle. The energy of the THz pulses was up to 2 *μ*J at pump laser energy of 700 *μ*J. Two parabolic mirrors were used to guide and to focus the THz pulses on the sample surface. The first mirror collected and collimated THz pulses emitted from the nonlinear crystal. The second one focused the THz beam into a spot with the diameter of 900 *μ*m. Such parameters provided a high amplitude of the THz electric field up to 300 kV/cm. The amplitude of the THz electric field was estimated based on the measurements of the pulse duration and pulse fluence. We have used about 3% of the output energy of the Cr:Forsterite laser beam reflected by a thin optical glass plate to probe the electric polarization in the studied medium. These optical probe pulses passed through a delay line and were focused into a spot of 200 *μ*m in diameter. After the sample, the fundamental optical radiation at the wavelength of 1240 nm was blocked and the SHG signal at the wavelength of 620 nm was transmitted by a bandpass filter (Δ*λ* = 10 nm). The SHG signal was detected by a Photo-Multiplier Tube in the current regime.

## Electronic supplementary material

Supplementary information

## References

[1] Nagaya K (2016). Ultrafast Dynamics of a Nucleobase Analogue Illuminated by a Short Intense X-ray Free Electron Laser Pulse. Physical Review X.

[2] Canton SE (2015). Visualizing the non-equilibrium dynamics of photoinduced intramolecular electron transfer with femtosecond X-ray pulses. Nature Communications.

[3] Bossini D, Belotelov VI, Zvezdin AK, Kalish AN, Kimel AV (2016). Magnetoplasmonics and Femtosecond Optomagnetism at the Nanoscale. ACS Photonics.

[4] De Jong JA (2012). Coherent control of the route of an ultrafast magnetic phase transition via low-amplitude spin precession. Physical Review Letters.

[5] Afanasiev D (2016). Control of the Ultrafast Photoinduced Magnetization across the Morin Transition in DyFeO_3_. Physical Review Letters.

[6] Vahaplar, K. *et al*. Ultrafast Path for Optical Magnetization Reversal via a Strongly Nonequilibrium State. *Physical Review Letters* **103**, doi:10.1103/PhysRevLett.103.117201 (2009). 10.1103/PhysRevLett.103.117201 19792396

[7] Landau, L. & Lifshitz, E. *Electrodynamics of continuous media* (Pergamon, Oxford, 1984).

[8] Rana DS (2009). Understanding the nature of ultrafast polarization dynamics of ferroelectric memory in the muitiferroic BiFeO_3_. Advanced Materials.

[9] Bhattacharjee, S., Rahmedov, D., Wang, D., Íñiguez, J. & Bellaiche, L. Ultrafast switching of the electric polarization and magnetic chirality in BiFeO_3_ by an electric field. *Physical Review Letters* **112**, doi:10.1103/PhysRevLett.112.147601 (2014). 10.1103/PhysRevLett.112.147601 24766014

[10] Brekhov KA (2015). Photoinduced dynamics and femtosecond excitation of phonon modes in ferroelectric semiconductor Sn_2_P_2_S_6_. JETP Letters.

[11] Miyamoto T, Yada H, Yamakawa H, Okamoto H (2013). Ultrafast modulation of polarization amplitude by terahertz fields in electronic-type organic ferroelectrics. Nature Communications.

[12] Yamakawa H (2016). Novel electronic ferroelectricity in an organic charge-order insulator investigated with terahertz-pump optical-probe spectroscopy. Scientific Reports.

[13] Kampfrath T, Tanaka K, Nelson Ka (2013). Resonant and nonresonant control over matter and light by intense terahertz transients. Nature Photonics.

[14] Katayama I (2012). Ferroelectric Soft Mode in a SrTiO_3_ Thin Film Impulsively Driven to the Anharmonic Regime Using Intense Picosecond Terahertz Pulses. Physical Review Letters.

[15] Qi T, Shin Y-H, Yeh K-L, Nelson KA, Rappe AM (2009). Collective Coherent Control: Synchronization of Polarization in Ferroelectric PbTiO_3_ by Shaped THz Fields. Physical Review Letters.

[16] Cornet M, Degert J, Abraham E, Freysz E (2014). Terahertz-field-induced second harmonic generation through Pockels effect in zinc telluride crystal. Optics Letters.

[17] Hruszkewycz, S. O. *et al*. Imaging local polarization in ferroelectric thin films by coherent X-ray bragg projection ptychography. *Physical Review Letters* **110**, doi:10.1103/PhysRevLett.110.177601 (2013). 10.1103/PhysRevLett.110.177601 23679778

[18] Schmising CVK, Bargheer M, Woerner M, Elsaesser T (2008). Real-time studies of reversible lattice dynamics by femtosecond X-ray diffraction. Zeitschrift fur Kristallographie.

[19] Oguz Er, A., Chen, J. & Rentzepis, P. M. Ultrafast time resolved x-ray diffraction, extended x-ray absorption fine structure and x-ray absorption near edge structure. *Journal of Applied Physics* **112**, doi:10.1063/1.4738372 (2012).

[20] Jiang AQ, Scott JF, Lu H, Chen Z (2003). Phase transitions and polarizations in epitaxial BaTiO_3_/SrTiO_3_ superlattices studied by second-harmonic generation. Journal of Applied Physics.

[21] Sherstyuk NE (2015). Optical Second Harmonic Generation Microscopy for Ferroic Materials. Ferroelectrics.

[22] Grishunin KA, Brekhov KA, O. V. S (2015). The study of the nature of the local optical inhomodeneities induced by ultrashort laser pulses, in the crystal of the ferroelectric-semiconductir Sn_2_P_2_S_6_. Russian Technological Journal.

[23] Fong DD, Thompson C (2006). *In Situ* Synchrotron X-Ray Studies of Ferroelectric Thin Films. Annual Review of Materials Research.

[24] Ejdrup T (2009). Picosecond time-resolved laser pump/X-ray probe experiments using a gated single-photon-counting area detector. Journal of Synchrotron Radiation.

[25] Grübel, S. *et al*. Ultrafast x-ray diffraction of a ferroelectric soft mode driven by broadband terahertz pulses. *arXiv* 1–5, 1602.05435 (2016).

[26] Chen F (2015). Ultrafast Terahertz Gating of the Polarization and Giant Nonlinear Optical Response in BiFeO_3_ Thin Films. Advanced Materials.

[27] Shirokov VB, Biryukov SV, Mukhortov VM, Yuzyuk YI (2011). Polarization of thin barium-strontium titanate films by an external electric field. Technical Physics.

[28] Mishina E, Sherstyuk N, Golovko Y, Muhortov V, Sigov A (2007). Polarization switching in ferroelectric thin films studied by optical second harmonic generation. Integrated Ferroelectrics.

[29] Wang F (1999). Calculation of the electro-optical and nonlinear optical coefficients of ferroelectric materials from their linear properties. Physical Review B.

[30] Veithen, M., Gonze, X. & Ghosez, P. First-principles study of the electro-optic effect in ferroelectric oxides. *Physical Review Letters* **93**, doi:10.1103/PhysRevLett.93.187401 (2004). 10.1103/PhysRevLett.93.187401 15525206

[31] Gopalan V, Mitchell TE (1999). *In situ* video observation of 180° domain switching in LiTaO_3_ by electro-optic imaging microscopy. Journal of Applied Physics.

[32] Mishina ED (2003). Domain orientation in ultrathin (Ba,Sr)TiO_3_ films measured by optical second harmonic generation. Journal of Applied Physics.

[33] Miller RC, Kleinman DA, Savage A (1963). Quantitative Studies of Optical Harmonic Generation in CdS, BaTiO_3_, and KH_2_PO_4_ Type Crystals. Physical Review Letters.

[34] Jiang AQ, Lee HJ, Hwang CS, Scott JF (2012). Sub-picosecond processes of ferroelectric domain switching from field and temperature experiments. Advanced Functional Materials.

[35] Mukhortov VM, Golovko YI, Tolmachev GN, Klevtzov AN (2000). The synthesis mechanism of complex oxide films formed in dense RF–plasma by reactive sputtering of stoichiometric targets. Ferroelectrics.

[36] Yuzyuk YI (2007). Influence of the growth mechanism and thermoelastic stresses on the lattice dynamics of heteroepitaxial films of barium strontium titanate. Physics of the Solid State.

[37] Anokhin AS, Razumnaya AG, Yuzyuk YI, Golovko YI, Mukhortov VM (2016). Phase transitions in barium-strontium titanate films on MgO substrates with various orientations. Physics of the Solid State.

[38] Padilla J, Zhong W, Vanderbilt D (1996). First-principles investigation of 180° domain walls in BaTiO_3_. Physical Review B.

[39] Vicario C (2015). High efficiency THz generation in DSTMS, DAST and OH1 pumped by Cr:forsterite laser. Optics Express.

