# Supplementary material for: THz Electric Field-Induced Second Harmonic Generation in Inorganic Ferroelectric
Source: Sci Rep. 2017 Apr 6;7:687. doi: 10.1038/s41598-017-00704-9 (PMC5429639; doi:10.1038/s41598-017-00704-9)
Supplement: Supplementary file 1 — Supplementary information [file 41598_2017_704_MOESM1_ESM.pdf]

THz Electric Field-Induced Second Harmonic Generation in Inorganic Ferroelectric

Kirill A. Grishunin[1,*], Nikita A. Ilyin[1], Natalia E. Sherstyuk[1], Elena D. Mishina[1], Alexey Kimel[1,2], Vladimir M. Mukhortov[3], Andrey V. Ovchinnikov[4], Oleg V. Chefonov[4], and Mikhail B. Agranat[4]

[1]Moscow Technological University, MIREA, Vernadsky Ave. 78, 119454, Moscow, Russia
[2]Radboud University Nijmegen, Institute for Molecules and Materials, 6525 AJ, Nijmegen, The Netherlands
[3]Southern Scientific Center of Russian Academy of Sciences, Chehova 41, Rostov-on-Don, 344006, Russia
[4]Joint Institute for High Temperatures of Russian Academy of Sciences (JIHT) Izhorskaya st. 13 Bd.2, 125412, Moscow, Russia
[*]grishunin@mirea.ru

Supplementary information

Details of computation

In the frame of crystallographic axes the electric polarization equals to $\vec{P} = \hat{\chi}\vec{E}_\omega\vec{E}_\omega$. In the laboratory frame mutual orientation of the wave polarizations (characterized by angle φ) and crystallographic axes as well as the angle of incidence θ_0 give more complex expressions.

In particular, the χ-tensor components should be transformed from the crystallographic frame $X_cY_cZ_c$ to the surface frame $X'Y'Z'$ (for our sample connected with (001) crystallographic face for unpertubed sample and (100) or (010) while THz in-plane field is applied) and then to the laboratory frame $X_LY_LZ_L$ by standard Euler transformations.

For our sample it results in the following expressions for non-linear optical polarization.

For the electric polarization aligned along the [001] crystallographic orientation nonlinear polarization at second harmonic wavelength can be written as:

$$
\begin{aligned}
P_x^{001} &= f_x f_z \chi_{xxz} \cos^2\varphi \sin 2\theta_0 \left(E_{\omega 0}\right)^2, \\
P_y^{001} &= f_y f_z \chi_{xxz} \sin 2\varphi \sin\theta_0 \left(E_{\omega 0}\right)^2, \\
P_z^{001} &= f_y^2 \chi_{xxz} \sin^2\varphi + \cos^2\varphi \left(f_x^2 \chi_{xxz} \cos^2\theta_0 + f_z^2 \chi_{zzz} \sin^2\theta_0\right) \left(E_{\omega 0}\right)^2,
\end{aligned}
$$

where $E_{\omega 0}$ is the amplitude of the input probe optical field, f_x, f_y and f_z are the Fresnel factors for X_L, Y_L and Z_L oriented input probe optical field.

For the electric polarization aligned along the [100] crystallographic orientations expressions for nonlinear polarization are:

$$
\begin{aligned}
P_x^{100} &= \left(-f_{1y}^2 \chi_{xxz} \sin^2\varphi - \left(f_{1x}^2 \chi_{xxz} \cos^2\theta_0 + f_{1z}^2 \chi_{xxz} \sin^2\theta_0\right) \cos^2\varphi\right) \left(E_{\omega 0}\right)^2, \\
P_y^{100} &= f_{1x} f_{1y} \chi_{xxz} \sin 2\varphi \cos\theta_0 \left(E_{\omega 0}\right)^2, \\
P_z^{100} &= f_{1x} f_{1z} \chi_{xxz} \cos^2\varphi \sin 2\theta_0 \left(E_{\omega 0}\right)^2.
\end{aligned}
$$

For the electric polarization aligned along the [010] crystallographic orientations expressions for nonlinear polarization are:

$$
\begin{aligned}
P_x^{010} &= f_{1x} f_{1y} \chi_{xxz} \sin 2\varphi \cos\theta_0 \left(E_{\omega 0}\right)^2, \\
P_y^{010} &= \left(-f_{1y}^2 \chi_{zzz} \sin^2\varphi - \chi_{xxz} \left(f_{1x}^2 \cos^2\theta_0 + f_{1x}^2 \sin^2\theta_0\right) \cos^2\varphi\right) \left(E_{\omega 0}\right)^2, \\
P_z^{010} &= f_{1x} f_{1z} \chi_{xxz} \sin 2\varphi \sin\theta_0 \left(E_{\omega 0}\right)^2.
\end{aligned}
$$

Fresnel factors

Fresnel factors for fundamental frequency:

$$f_{1x} = \frac{2\cos\theta_0}{\varepsilon_1\cos\theta_0+\sqrt{\varepsilon_1-sin^2\theta_0}}$$

$$f_{1y} = \frac{2\cos\theta_0}{\cos\theta_0+\sqrt{\varepsilon_1-sin^2\theta_0}},$$

$$f_{1z} = \frac{2\sqrt{\varepsilon_1-sin^2\theta_0}}{\varepsilon_1\cos\theta_0+\sqrt{\varepsilon_1-sin^2\theta_0}},$$

Fresnel factors for doubled frequency:

$$f_{2x} = \frac{4\pi\sqrt{\varepsilon_2-sin^2\theta_0}}{\left(\varepsilon_2\cos\theta_0+\sqrt{\varepsilon_2-sin^2\theta_0}\right)\left(\sqrt{\varepsilon_1-sin^2\theta_0}+\sqrt{\varepsilon_2-sin^2\theta_0}\right)},$$

$$f_{2y} = \frac{4\pi\left(-\sqrt{\varepsilon_1-sin^2\theta_0}+\sqrt{\varepsilon_2-sin^2\theta_0}\right)}{(\varepsilon_2-\varepsilon_1)\left(\cos\theta_0+\sqrt{\varepsilon_2-sin^2\theta_0}\right)},$$

$$f_{2z} = \frac{4\pi\sin\theta_0}{\left(\varepsilon_2\cos\theta_0+\sqrt{\varepsilon_2-sin^2\theta_0}\right)\left(\sqrt{\varepsilon_1-sin^2\theta_0}+\sqrt{\varepsilon_2-sin^2\theta_0}\right)}.$$

where $\theta_0 = 23^\circ$ is the incidence angle of the probe beam, $\varepsilon_1, \varepsilon_2$ are electric permittivity for the fundamental and doubled frequency, respectively.

After substituting the numerical values of permittivity ($\varepsilon_1 = 5.4$ and $\varepsilon_2 = 6.55$ for fundamental and doubled frequency, respectivetly), it gives:

$$f_{1x} = 0.253, \quad f_{2x} = 0.771,$$
$$f_{1y} = 0.573, \quad f_{2y} = 0.756,$$
$$f_{1z} = 0.631, \quad f_{2z} = 0.12.$$

Optical rotation, No THz field (for Fig. 1(b))

Without the Terahertz field the electric polarization of the film is oriented in the [001] direction. The SHG signal is determined by orientation of the film and the SHG intensity for the Pout and the Sout polarizations, can be written as

$$\begin{aligned} I^{p-out}_{2\omega,001} &= \left(A^{p-out}_{001}\cos^2\varphi + B^{p-out}_{001}\sin^2\varphi\right)^2 (E_{\omega 0})^4 = \\ &= sin^2\theta_0 \left(cos^2\varphi\left(f_{1x}\left(2f_{1z}f_{2x}+f_{1x}f_{2z}\right)\chi_1\cos^2\theta_0 + f^2_{1z}f_{2z}\chi_2\sin^2\theta_0\right) + f^2_{1y}f_{2z}\chi_1\sin^2\varphi\right)^2 (E_{\omega 0})^4 \end{aligned} \quad (1)$$

$$I^{s-out}_{2\omega,001} = C^{s-out}_{001}\sin^2 2\varphi\,(E_{\omega 0})^4 = f^2_{1y}f^2_{1z}f^2_{2y}\chi^2_1\sin^2\theta_0\sin^2 2\varphi\,(E_{\omega 0})^4, \quad (2)$$

where A^{p-out}_{001}, B^{p-out}_{001} and A^{p-out}_{001} are linear combinations of the Fresnel factors and tensor components of the nonlinear susceptibility; φ is the angle of the polarization of the incoming light with respect to the [100] axis.

After substituting the numerical values of Fresnel factors and angle of incidence it gives:

$$I^{p-out}_{2\omega,001} = \left((0.084\chi_1 + 0.003\chi_2)\cos^2\varphi + 0.016\chi_1\sin^2\varphi\right)^2 (E_{\omega 0})^4, \quad (3)$$

$$I^{s-out}_{2\omega,001} = 0.012\chi^2_1\sin^2 2\varphi\,(E_{\omega 0})^4. \quad (4)$$

Coefficients equal to:

$$\begin{aligned} A^{p-out}_{001} &= 0.084\chi_1 + 0.003\chi_2, \\ B^{p-out}_{001} &= 0.016\chi_1, \\ C^{s-out}_{001} &= 0.012\chi^2_1. \end{aligned}$$

Optical rotation, THz field oriented along X_L (for Fig. 1(c))

When the in-plane electric field is applied at angle ψ respect to X_L axis, a part of the domains line up along the field. The net response will be defined by volume fractions of domains V^+_i and $V^-_i (i = x,y)$ $(i = x,y)$,where x,y denote the crystallographic axis along which the polarization is aligned, but "+" and "-" show the direction of the alignment. The differences of the fractions of the positively and the negatively oriented domains determines the electric field dependent contribution to the nonlinear optical polarization as $P_i = P^{001}_i + \Delta V_i P_i$, where $\Delta V_i = V^+_i - V^-_i$.

The volume contributions to the corresponding domain directions for any angle ψ of the applied THz E-field results in the following dependences:

$$\Delta V_x = \gamma\cos\psi,$$
$$\Delta V_y = \gamma\sin\psi,$$
$$\Delta V_z = \sqrt{1-\Delta V_x{}^2 - \Delta V_y{}^2},$$

where γ is the ratio of the fraction of in-plane switched domains to the fraction of [001]-oriented unswitched domains.

For the THz E-field oriented along the x-axis ([100] crystallographic direction), a rotation of the polarization of the incoming probe light results in the following dependencies

$$\begin{aligned} I_{2\omega}^{p-out} &= \left(\sqrt{1-\gamma^2}\left(A\left(\Delta V_x\right)\cos^2\varphi + B\left(\Delta V_x\right)\sin^2\varphi\right) - \gamma\left(C\left(\Delta V_x\right)\cos^2\varphi + D\left(\Delta V_x\right)\sin^2\varphi\right)\right)^2\left(E_{\omega 0}\right)^4 = \\ &= \left(\sqrt{1-\gamma^2}\sin\theta_0\left(cos^2\varphi\left(f_{1x}\left(2f_{1z}f_{2x}+f_{1x}f_{2z}\right)\chi_1\cos^2\varphi_0 + f_{1z}^2f_{2z}\chi_2\sin^2\theta_0\right) + f_{1y}^2f_{2z}\chi_1\sin^2\varphi\right) - \right. \\ &\left. -\gamma\cos\theta_0\left(\cos^2\varphi\left(f_{1x}^2f_{2x}\chi_2\cos^2\theta_0 + f_{1z}\left(f_{1z}f_{2x}+2f_{1x}f_{2z}\right)\chi_1\sin^2\theta 0\right) + f_{1y}^2f_{2x}\chi_1\sin^2\varphi\right)\right)^2\left(E_{\omega 0}\right)^4, \end{aligned} \tag{5}$$

$$\begin{aligned} I_{2\omega}^{s-out} &= \left(\sqrt{1-\gamma^2}E\left(\Delta V_x\right) - \gamma G\left(\Delta V_x\right)\right)^2\sin^2 2\varphi\left(E_{\omega 0}\right)^4 = \\ &= f_{2y}^2\left(\sqrt{1-\gamma^2}f_{1y}f_{1z}\chi_1\sin\theta_0 - \gamma f_{1x}f_{1y}\chi_1\cos\theta_0\right)^2\sin^2 2\varphi\left(E_{\omega 0}\right)^4. \end{aligned} \tag{6}$$

where $A\left(\Delta V_x\right)$, $B\left(\Delta V_x\right)$, $C\left(\Delta V_x\right)$, $D\left(\Delta V_x\right)$, $E\left(\Delta V_x\right)$ and $G\left(\Delta V_x\right)$ are linear combinations of the Fresnel factors and tensor components of the nonlinear susceptibility. All these coefficients are weighted in accordance with the fraction of a corresponding domain. Analogously, similar dependeces of the SHG intensity can be written for the case of THz field, oriented along the y axis. Such a field promotes formation of ΔV_y domains.

After substituting the numerical values of Fresnel factors and angle of incidence it gives:

$$\begin{aligned} I_{2\omega}^{p-out} &= \left(\sqrt{1-\gamma^2}\left(\left(0.0086\chi_1 + 0.003\chi_2\right)\cos^2\varphi + 0.04\chi_1\sin^2\varphi\right) - \right. \\ &\left. -\gamma\left(\left(0.046\chi_1 + 0.037\right)\cos^2\varphi + 0.23\chi_1\sin^2\varphi\right)\right)^2\left(E_{\omega 0}\right)^4, \end{aligned} \tag{7}$$

$$I_{2\omega}^{s-out} = \left(0.107\chi_1\sqrt{1-\gamma^2} - 0.101\chi_1\gamma\cos\varphi\sin\varphi\right)^2\sin^2 2\varphi\left(E_{\omega 0}\right)^4. \tag{8}$$

Coefficients equal to:

$$\begin{array}{l|l} A(\Delta V_x) = 0.0086\chi_1 + 0.003\chi_2, & D(\Delta V_x) = 0.23\chi_1, \\ B(\Delta V_x) = 0.04\chi_1, & E(\Delta V_x) = 0.107\chi_1, \\ C(\Delta V_x) = 0.046\chi_1 + 0.037, & G(\Delta V_x) = 0.101\chi_1. \end{array}$$

THz rotation, Optical field oriented along X_L (for Fig. 1(d))

Then for the optical probe polarization parallel to the x-axis ($\varphi = 0$):

$$\begin{aligned} I_{2\omega}^{p-out}\left(E_\Omega\right) &= \left(A_\Omega\sqrt{1-\gamma^2} - B_\Omega\gamma\cos\psi\right)^2\left(E_{\omega 0}\right)^4 = \\ &= \left(-\left(\gamma\cos\theta_0\cos\psi\left(f_{1x}^2f_{2x}\chi_2\cos^2\theta_0 + f_{1z}\left(f_{1z}f_{2x}+2f_{1x}f_{2z}\right)\chi_2\sin^2\theta_0\right)\right) + \right. \\ &\left. +\sqrt{1-\gamma^2}\sin\theta_0\left(f_{1x}\left(2f_{1z}f_{2x}+f_{1x}f_{2z}\right)\chi_1\cos^2\theta_0 + f_{1z}^2f_{2z}\chi_2\sin^2\theta_0\right)\right)^2\left(E_{\omega 0}\right)^4, \end{aligned} \tag{9}$$

$$I_{2\omega}^{s-out}\left(E_\Omega\right) = \left(C_\Omega\gamma\sin\psi\right)^2\left(E_{\omega 0}\right)^4 = f_{2y}^2\gamma^2\chi_1^2\left(f_{1x}^2\cos^2\theta_0 f_{1z}^2\sin^2\theta_0\right)^2\sin^2\psi\left(E_{\omega 0}\right)^4. \tag{10}$$

After substituting the numerical values of Fresnel factors and angle of incidence it gives:

$$I_{2\omega}^{p-out}\left(E_\Omega\right) = \left(-\gamma(0.049\chi_1 + 0.039\chi_2)\cos\psi + \sqrt{1-\gamma^2}\left(0.084\chi_1 + 0.0029\chi_2\right)\right)^2\left(E_{\omega 0}\right)^4, \tag{11}$$

$$I_{2\omega}^{s-out}\left(E_\Omega\right) = 0.008\gamma^2\chi_1^2\sin^2\psi\left(E_{\omega 0}\right)^4. \tag{12}$$

Coefficients equal to:

$$\begin{aligned} A_\Omega &= 0.084\chi_1 + 0.0029\chi_2, \\ B_\Omega &= 0.049\chi_1 + 0.039\chi_2. \end{aligned}$$

Fitting parameters (simultaneous fit)

In the simultaneous fit only 2 meaningful and 1 calibration fitting parameters were used giving

$$\chi_1 = 10.7 \pm 0.02,$$
$$\chi_2 = 3.1 \pm 0.9,$$
$$\gamma = 0.060 \pm 0.006.$$
